# Surgical Outcomes in Non-Transected and Partially Transected Peripheral Nerve Injuries

**DOI:** 10.3390/brainsci15111202

**Published:** 2025-11-07

**Authors:** Naveen Arunachalam Sakthiyendran, Karter Morris, Caroline J. Cushman, Evan J. Hernandez, Anceslo Idicula, Brendan J. MacKay

**Affiliations:** 1 Department of Neurosurgery, Boston University School of Medicine, Boston, MA 02118, USA; naveenar@bu.edu; 2 School of Medicine, Texas Tech University Health Sciences Center, Lubbock, TX 79430, USA; karter.morris@ttuhsc.edu (K.M.); caroline.cushman@ttuhsc.edu (C.J.C.); 3 Department of Orthopedic Surgery, University Medical Center, Texas Tech University Health Sciences Center, Lubbock, TX 79430, USA; evan.j.hernandez@ttuhsc.edu (E.J.H.); anceslo.idicula@ttuhsc.edu (A.I.); 4 Department of Health Sciences, College of Health Sciences, Rush University, Chicago, IL 60612, USA; 5 Hand and Microvascular Surgery, University Medical Center, Texas Tech University Health Sciences Center, Lubbock, TX 79430, USA

**Keywords:** nerve injury, median nerve, reconstruction

## Abstract

Background: Non-transected and partially transected peripheral nerve injuries (neuromas-in-continuity) are relatively common but understudied. Their optimal surgical management and expected outcomes remain unclear. We conducted a literature review of surgical repairs in such lesions and illustrate a case to guide decision-making. Systematic searches of PubMed and Google Scholar identified 70 eligible reports (Level I = 2, Level II = 5, Level III = 37, Level IV = 20, Level V = 4). Across studies, neurolysis of NAP-positive lesions often restored antigravity strength, while direct repair or grafting of nonconductive segments yielded meaningful recovery in ~75%. After neurolysis or reconstruction, ~77–92% of brachial plexus/axillary neuromas-in-continuity reached LSUHSC Grade ≥3. Median/ulnar lesions treated with neurolysis, biologic/vascularized coverage, or reconstruction showed reliable pain relief but variable sensory/motor recovery. Radial/PIN lesions improved in some series irrespective of NAPs. Earlier intervention, shorter gaps, distal sites, and younger age correlated with superior outcomes. Meanwhile, prolonged observation risking end-organ atrophy degraded results. Adjuncts such as electrical stimulation and wraps may aid reinnervation or reduce scarring, though high-quality evidence is limited. Conclusions: For non-transected and partially transected PNIs, a pragmatic approach emerges: Observe low-grade injuries with serial examinations. Explore early if recovery stalls (≈3–6 months). Use NAP-guided neurolysis for conductive lesions. Perform tension-free repair or grafting for nonconductive segments, adding anti-adhesive coverage when appropriate. Standardized reporting and prospective trials are needed to refine timing, technique selection, and patient-reported outcomes.

## 1. Introduction

Peripheral nerve injuries (PNIs) are a significant cause of long-term morbidity and functional disability, particularly in young adults and working populations [1]. Although transected nerves often prompt immediate surgical intervention, non-transected and partially transected nerve injuries are frequently encountered in clinical practice and present a unique diagnostic and therapeutic challenge [2]. These injuries may result from blunt trauma, traction, compression, or iatrogenic causes, and often demonstrate variable disruption of neural and connective tissue architecture without complete discontinuity [1]. Because axonal integrity may be partially preserved in these injuries, spontaneous recovery is possible in some cases. However, others may progress to neuroma formation, Wallerian degeneration, and chronic denervation, with poor functional outcomes if untreated [2]. Accurate diagnosis, timely surgical decision-making, and an understanding of which patients will benefit from operative repair remain poorly defined in this subset of nerve injuries.

Historically, much of the peripheral nerve literature has focused on completely transected nerves, leading to a relative underrepresentation of non-transected and partially transected injuries in clinical studies. Consequently, there is less consensus regarding the indications, techniques, and outcomes of surgical intervention in these cases. Furthermore, the development of advanced intraoperative tools, including high-resolution ultrasound, intraoperative nerve stimulation, and intraoperative neurophysiological monitoring, has increased the ability to identify subclinical or occult nerve injuries, prompting reconsideration of long-held conservative approaches. This review synthesizes the existing literature on the outcomes of nerve repair in non-transected and partially transected peripheral nerves, evaluates the clinical contexts in which surgery may be beneficial, and highlights areas in need of further study. By improving our understanding of this often-overlooked subset of PNIs, we aim to provide clinicians with a more nuanced framework for evaluating and managing these complex injuries.

## 2. Methods

A literature review was conducted using PubMed and Google Scholar research databases and in forward and backward citations (from database inception to January 2025) using the following keywords: “peripheral nerve injury”, “nerve repair”, “axillary or median or ulnar or radial or digital”, “non-transected”, “partial-transection”, or “lesion in-continuity”. We limited the search to English-language articles and included clinical studies (of any level of evidence) as well as relevant case series and case reports that reported outcomes of surgical intervention on incomplete nerve injuries. Articles focusing exclusively on complete nerve transections or on chronic compressive neuropathy without acute injury were excluded unless they provided segregated data on partial injuries. From the search results, 70 publications meeting these criteria were selected for detailed review and data extraction. These 70 studies were categorized by level of evidence according to the *Journal of Hand Surgery* guidelines. Specifically, our review included 2 Level I studies, 5 Level II studies, 37 Level III studies, 20 Level IV studies, 4 Level V (expert opinion or case report) articles, and 2 animal studies. We extracted information on patient populations, injury types, surgical treatments, and outcomes from each study, aiming to synthesize overall success rates and prognostic factors. No meta-analysis was performed given the heterogeneity of reports; instead, results are reported narratively and compared qualitatively across studies.

### Case Illustration

Presentation and Mechanism of Injury: A 48-year-old right-hand-dominant male sustained a high-voltage electrocution after grasping a 13,000-volt transformer with his right hand. He presented with burn injuries to the right hand and an immediate, dense sensory deficit in the median and ulnar nerve distributions, accompanied by ulnar clawing, consistent with a mixed sensorimotor peripheral nerve injury.

**Figure 1:**
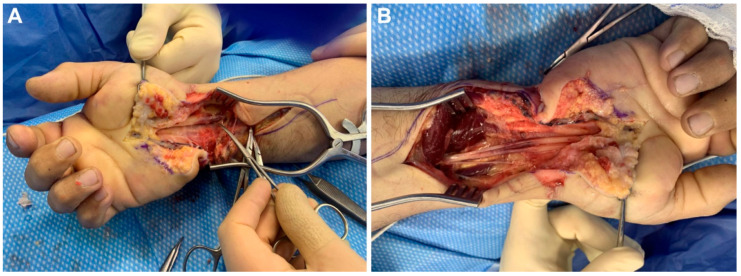
High-voltage electrocution causing lesion-in-continuity of the median and ulnar nerves—operative findings and management. (**A**) Volar wrist/hand exposure after same-day presentation shows swollen, ecchymotic median nerve within the carpal tunnel and distal forearm following a 13,000 V injury. Carpal tunnel release and external neurolysis were performed to decompress potential entrapment sites and assess fascicular continuity/viability. (**B**) Post-decompression view demonstrating an in-continuity, contused median nerve (no transection). Affected segment was wrapped with an Avive^®^ allograft to provide a biologic anti-adhesive interface, modulate local inflammation, and limit perineural fibrosis that could impede regeneration.

On arrival, the patient exhibited profound paresthesia/numbness in the median- and ulnar-innervated digits and intrinsic hand weakness reflected clinically by clawing of the ulnar digits. Given the mechanism (thermal + electrical), physical findings, and high risk of progressive intraneural coagulative injury, a lesion-in-continuity of both the median and ulnar nerves was suspected (Figure 1A). Electrodiagnostic testing was not reported at this acute stage, and the decision was made to proceed with urgent surgical exploration to decompress potential sites of entrapment, evaluate intraneural viability, and prevent secondary ischemic/fibrotic change.

Operative Management: The patient underwent same-day exploration with carpal tunnel release and neurolysis in the distal forearm. Intraoperatively, the median nerve demonstrated a distinctly ecchymotic segment but remained structurally in continuity, consistent with a thermally/electrically contused fascicular architecture rather than a complete transection. The affected segment was wrapped with a 2 × 4 cm Avive^®^ umbilical cord allograft to (1) provide a biologic, anti-adhesive interface, (2) modulate the local inflammatory milieu, and (3) potentially reduce perineural fibrosis that could impede axonal regeneration (Figure 1B). The ulnar nerve underwent external neurolysis; no segmental resection or grafting was performed for either nerve, reflecting the intraoperative judgment that recovery potential existed and that a resection/coaptation would sacrifice remaining viable fascicles in this in-continuity context.

Postoperative Course and Functional Recovery: At 11 months postoperatively, the patient reported progressive sensory improvement but persistent, intermittent numbness localized to the index (IF) and ring fingers (RF). Importantly, he had no pain, was sensate overall, and able to move all digits, though he continued to experience functional limitation with power grip and could not achieve a composite fist. Quantitatively, grip strength on the injured side measured 30 lbs compared with 80 lbs contralaterally, and key pinch strength measured 6 lbs vs. 12 lbs on the contralateral side. Despite these measurable deficits, the absence of neuropathic pain and the presence of improving—albeit incomplete—sensation argue that axonal regeneration occurred, but with incomplete reinnervation and/or end-organ changes limiting full strength return. Formal electrodiagnostic testing (EMG/NCS) was not performed acutely; however, chronic follow-up data would further elucidate reinnervation extent and conduction recovery compared to the contralateral side. Future cases should integrate longitudinal EMG/NCS assessments to objectively quantify axonal continuity, motor unit recruitment, and conduction velocity restoration, complementing grip and pinch strength metrics.

Ethics Approval: Per the TTUHSC Human Research Protection Program Manual, IRB review and informed consent are required when conducting human subjects research involving three or more individuals. Since this project involved a single class activity and does not meet that threshold, IRB approval and consent were not required. However, written informed consent was obtained for publication of the case details and images.

## 3. Review

### Classification and Mechanics of Injury

**Figure 2:**
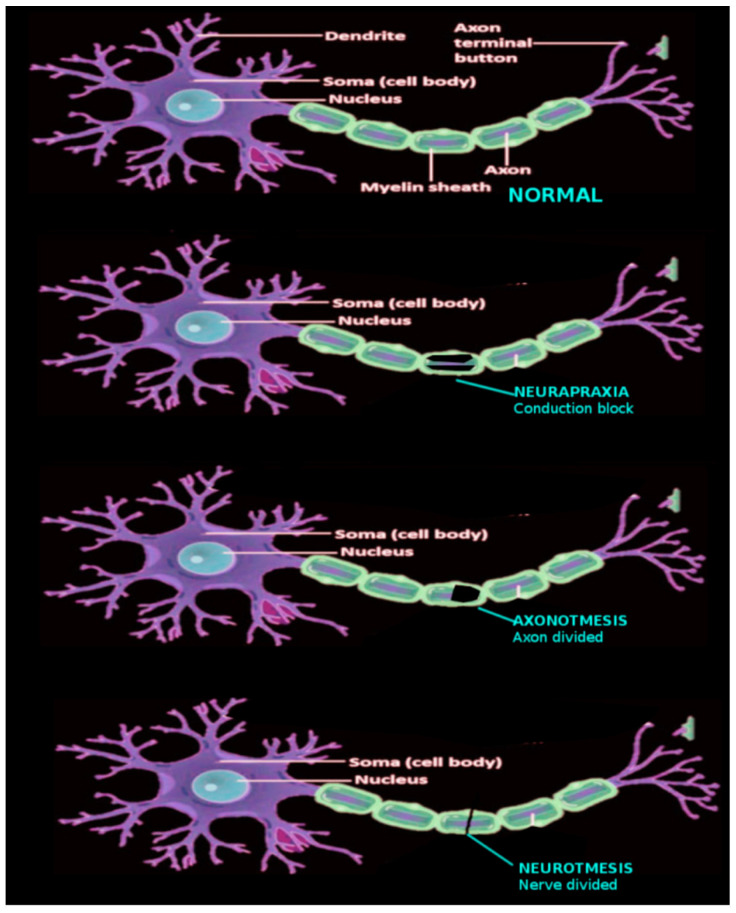
**Classification of Nerve Injuries.** available at https://www.ncbi.nlm.nih.gov/books/NBK549848/figure/article-25742.image.f2/ (accessed on 24 August 2025) and distributed under the terms of the Creative Commons Attribution 4.0 International License (https://creativecommons.org/licenses/by/4.0/, accessed on 24 August 2025).

Historically, nerve injuries have been categorized by the extent of axonal and connective tissue damage. In 1943, Sir Herbert Seddon proposed the first formal classification system, dividing nerve injuries into three categories based on presence of demyelination and extent of damage to the axons and connective tissues of the associated nerve [1,2,3,4,5,6]. Neurapraxia is defined by focal demyelination without damage to the axons or the connective tissues. Axonotmesis involves direct damage to the axons with focal demyelination and maintained continuity of connective tissues. Lastly, neurotmesis is full transection of the axons and connective tissue layers with complete discontinuity of the nerve. Any of these three levels of injury may result in decreased or absent neural conduction, and subsequent muscle weakness or paralysis (Figure 2).

In 1951, Sydney Sunderland expanded Seddon’s scheme into a five-grade system, subdividing partial injuries by the degree of connective tissue disruption. Sunderland grade I corresponds to neurapraxia; Grades II–IV represent increasing severity of axonotmesis (axonal injury with endoneurial, perineurial, and epineurial damage respectively); and Grade V represents neurotmesis [7]. This classification highlights that “in-continuity” nerve lesions (Grades I–IV) retain some structural continuity, whereas Grade V injuries do not. (Table 1).

Within this structural framework, mechanisms of peripheral nerve injury often anticipate the Sunderland grade and guide early management. PNIs can occur by a variety of mechanisms, including chronic compression, acute compression (crush), laceration, or avulsion. Complete transections (Sunderland V, neurotmesis) most often result from sharp lacerations (e.g., knife, glass, shrapnel) and cause immediate loss of nerve continuity [8]. Paradoxically, although these injuries are severe, their management is relatively straightforward: the primary surgical decision is how to bridge the gap (direct repair versus graft) based on defect length and tissue condition.

Given that neurapraxia does not involve transection of the axon, these injuries may be considered “non-transected”. The majority of non-transected (neurapraxia, Grade I) injuries occur due to compression injuries (e.g., carpal tunnel syndrome (CTS)), or entrapment of nerves in locations with narrow anatomical openings (e.g., cubital tunnel syndrome) [1]. Patients have transient conduction block and demyelination, but spontaneous recovery is expected once the compressive cause is relieved.

“Partially-transected” nerve injuries may essentially be considered a form of axonotmesis, as these injuries involve damage to the axon, making them more severe than neurapraxia, but without the complete transection observed in neurotmesis [9]. Partially-transected nerves (axonotmesis, Grades II–IV) are most often caused by stretch or crush injuries, such as a blow from a blunt object or crush by a surgical clamp [1]. Of note, the compromised portion of a partially transected nerve may regenerate on its own [9]. However, if the damaged proximal neuron fails to reach the distal target with organized proliferation along the injured segment, Wallerian degeneration and neuroma formation becomes a concern.

When natural regeneration is unsuccessful and an intact, lengthwise portion continues to transmit signals, the remaining defect is referred to as a neuroma-in-continuity [10]. Neuromas-in-continuity can cause pain, hypersensitivity, sensory deficits, or impaired motor function, and may require surgical intervention to improve clinical outcomes. Several studies have indicated numerous treatment modalities for neuroma, although there is no consensus on the optimal algorithm for neuroma management [11,12].

## 4. Diagnosis of Peripheral Nerve Injuries

Accurate early diagnosis of a nerve injury’s severity and extent is critical yet challenging. Clinical history and physical examination (motor/sensory testing) guide initial assessment. Electrodiagnostic studies, including electromyography (EMG) and nerve conduction studies (NCS), are traditional diagnostic tools. Needle EMG can detect signs of denervation in muscle (e.g., fibrillations, reduced motor unit recruitment), while NCS measure conduction velocity and amplitude in motor and sensory fibers. These tests help distinguish demyelination from axon loss and estimate injury severity. However, a key limitation is timing: electrodiagnostic changes indicative of denervation often do not manifest until 3–4 weeks post-injury; in fact, one recent study showed that the absence of motor unit potentials on EMG has high predictive value for severe injury only after about 4 months, which can be too late for optimal surgical repair [2]. This initial period of time has shown to be critical in the prognosis of the injured nerves as motor unit potentials are lost and Wallerian degeneration ensues [2]. The delay in diagnostic clarity means that the critical early window for nerve repair may be missed if one waits for confirmatory EMG/NCS results.

Another significant limitation that clinicians face in the diagnosis and further evaluation of nerve injuries is that there are various cellular, metabolic, or inflammatory processes that are critical in determining treatments beyond just the disruption of anatomical structures characterized by the Sunderland classification. The role of inflammation, for example, further complicates the physiology surrounding nerve regeneration as inflammation is an essential factor that promotes normal peripheral nerve regeneration [13]. However, excessive or prolonged inflammation can lead to fibrosis and chronic pain. Evidence suggests that modulating the immune/inflammatory environment might enhance nerve regeneration [14], underscoring that factors like inflammation, Schwann cell response, and the molecular microenvironment can critically affect outcomes even when the anatomical lesion appears similar.

Advanced imaging can supplement diagnosis. Magnetic Resonance Neurography (MRN) uses high-resolution MRI sequences to visualize nerve anatomy and detect pathologies like nerve discontinuity, neuroma formation, or entrapment. MRN has shown promise in differentiating injured vs. healthy nerves and may help determine if surgery is warranted in cases of equivocal injury [15]. High-resolution ultrasound (US) is another valuable tool: it allows real-time visualization of peripheral nerves and can identify nerve enlargement, loss of continuity, neuromas, or internal nerve fascicle structure abnormalities [15]. Ultrasound is especially useful for superficial nerves and intraoperative evaluation–studies have demonstrated that ultrasound can determine whether a nerve is completely or incompletely severed and detect neuromas or scar tissue around nerves [15]. Compared to MRI, ultrasound is more accessible and cost-effective, though MRI better visualizes deep structures and multiple injury sites [15].

## 5. Treatment and Outcomes for Non-Transected and Partially Transected Nerve Injuries

Treatment strategies for non-transected or partially transected PNIs range from conservative management to various surgical interventions. Broadly, options include: (1) nonoperative management, (2) conventional surgical repair (neurolysis or direct repair/grafting), (3) advanced or adjunctive surgical techniques (e.g., nerve wraps, flaps, nerve transfers), or (4) combined approaches [16,17]. Common outcome measures reported in studies include anatomical assessments (e.g., histological axon counts, number of reinnervated motor end-plates), electrophysiological improvements (EMG/NCS findings), and functional recovery via sensory tests (e.g., two-point discrimination) or motor strength (grip strength, MRC grades). Patient-reported outcomes like pain relief (e.g., visual analog scale) and disability scores (e.g., DASH/QuickDASH) are also frequently documented [1].

Some patients who suffer a PNI may delay seeking treatment of any kind. Novak et al. evaluated the Disabilities of the Arm, Shoulder, and Hand (QUICKDASH) scores of 84 patients (mean age of 39 years) who presented to a nerve surgeon at least 6 months (mean time 38 months) after having suffered a PNI. Substantial long-term functional disability (high QUICKDASH score) was found in this group, especially among the patients with higher pain, older age, and brachial plexus injuries. These results indicate that patients who delay seeking treatment for PNI could be at risk for decreased health-related quality of life [18].

### 5.1. Nonoperative Treatment

Standard nonoperative treatment generally entails observation with EMG and NCS [16]. In some cases, pain management with medication, physical therapy rehabilitation exercises, and/or bracing are initiated prior to any decisions regarding surgical versus nonsurgical intervention [16].

Although the conservative approach has some advantages—especially for many lesions in-continuity—there is a risk of poor outcomes secondary to treatment delay [19]. For example, Omer performed a prospective study of upper extremity PNIs and found that of the 648 nerve lesions in-continuity observed, 454 (70%) demonstrated spontaneous recovery. The markers of good clinical recovery were based on motor recovery, defined as a return of function that demonstrated nerve regeneration and the ability to move the extremity against resistance, and sensory recovery, which was specified as the patient’s ability to detect within a 20 mm distance on a two-point discrimination (2PD) test. Time to spontaneous recovery from the initiation of treatment was evaluated based on mechanism of injury and varied from 3–9 months for gunshot wounds, and 1–4 months for fracture-dislocations. However, in this report, Omer did not provide data regarding motor or sensory function of the injured nerves/extremities prior to treatment, leaving some ambiguity as to the initial severity of the PNIs [20].

While this preliminary study was promising, more recent data underscore the potential need for surgery for more clinically significant improvement. In a retrospective study of 311 PNIs, Wang et al. compared 53 patients managed nonoperatively versus 258 who underwent surgery. Prior to treatment, the Modified British Medical Research Council System (graded 0 to 5) showed the surgical intervention group to have significantly lower motor (1.19 vs. 2.23) and sensory examination scores (2.06 vs. 2.28) when compared with the nonoperative group. This suggests that milder injuries were selected into the nonoperative cohort and tended to recover well, whereas more severe injuries required surgery. In other words, if a nerve injury is truly low-grade (neurapraxia or limited axonotmesis), observation can yield outcomes comparable to surgery, but higher-grade partial injuries often cannot be expected to recover fully without intervention [17].

Additionally, given that conservative approaches inherently involve delays in active treatment, waiting too long for spontaneous recovery can allow irreversible end-organ changes (muscle atrophy, end-plate fibrosis) and reduce the chances of surgical success later, if warranted. Hence, during the “wait-and-see” period, careful serial exams are essential. Signs of reinnervation (fibrillations resolving on EMG, emerging voluntary muscle activity, advancing Tinel’s sign, improving sensory thresholds) indicate regeneration [21,22]. If no signs of recovery appear in a given timeframe (often 3–6 months depending on injury level), one should strongly consider surgical exploration to prevent permanent deficits [23].

### 5.2. Surgical Management

If a PNI fails nonoperative treatment, surgical treatment may be indicated [19]. Repair for stump (end) neuromas almost always consists of complete excision. Neuromas-in-continuity, on the other hand, may be treated with intraneural neurolysis, which consists of dissecting scar tissue from around the injured nerve segment, and the selective removal of neuroma. Another option in place of intraneural neurolysis is to bypass the neuroma altogether through the use of a nerve graft [24]. Either of these procedures in themselves may be sufficient to restore nerve action potentials (NAP) and function/sensation [25]. However, in the event that no NAP is recorded following neurolysis, further repair is required [26]. This may be done through coaptation, preferably through a direct end-to-end repair technique that entails excising the non-functional nerve segment and then rejoining the remaining proximal and distal nerve segments [27]. When approximation of the primary nerve ends would cause significant tension, grafting is indicated [1].

Multiple reports have listed factors that improve outcomes for the surgical repair of PNIs, for example: early repairs are better than late; nerve coaptation is better than nerve grafts; young patients do better than older patients; distal repair is better than proximal repair; and short grafts do better than long grafts [19,28]. These principles were largely informed by historical understanding of complete transections. In these cases, the literature indicates that gap length and mechanism of injury often should determine the appropriate course of treatment [29]. However, literature describing outcomes of various treatment outcomes for incomplete or non-transected nerve injuries is limited.

## 6. Nerve-Specific Evidence and Details

### 6.1. Brachial Plexus and Axillary Nerve

High-energy trauma can produce complex brachial plexus injuries where elements of the plexus are partially damaged and form neuromas-in-continuity. In one study of 57 brachial plexus neuromas-in-continuity, 26 nerves had positive NAPs and were treated by neurolysis, and 31 lesions absent for NAP were repaired using either suture (9) or graft (22) technique. Outcomes were reported as elements recovering to a Louisiana State University Health Sciences Center (LSUHSC) Grade 3 or better. An LSUHSC grade is calculated by assigning each element a postoperative recovery grade (0–5), these grades are then summed and averaged to determine the overall grade. Patients treated by neurolysis, suture, or graft achieved at least Grade 3 recovery in 92%, 78%, and 77% of cases respectively [30]. If the lesion was non-conductive and required reconstruction, outcomes were slightly worse but still about three-quarters of patients attained meaningful function.

The axillary nerve, a branch of the posterior cord supplying the deltoid and teres minor, is often stretched or contused in shoulder injuries (e.g., shoulder dislocations, traction injuries). Many axillary nerve injuries in athletes are neurapraxias that recover with nonoperative management. This may result in axillary palsy and deltoid paralysis. In a retrospective study of 99 axillary neuromas-in-continuity, 30 cases of lesions with NAP were treated by neurolysis and achieved a mean LSUHSC Grade 4.0. In lesions absent for NAP, three were repaired by suture, and sixty-six were treated by surgical graft, achieving mean Grades of 3.8 and 3.7 respectively. The mean time from injury to surgery in that series was 6.5 months, with follow-up ~4 years. All groups showed relatively good recovery (averaging Grade ~3.7–4.0 out of 5), again highlighting that many patients regained antigravity muscle strength [31].

A study by Lee et al. evaluated 26 sports injury-related axillary nerve lesions in-continuity with positive NAP after six–eight months of nonoperative treatment (e.g., monthly evaluation by EMG, changing pitching mechanics) had failed. Preoperatively, injuries were stratified by LSUHSC grade: those with partial deficits (Grade 1–2) underwent neurolysis, while complete palsies (Grade 0) were managed with neurolysis in three cases and surgical repair (suture in two cases, graft in twelve cases) in the rest. Postoperative outcomes were favorable: the neurolysis group improved to a mean Grade 4.2, and even the repair groups (suture or graft) achieved Grades in the 3.0–4.0 range on average. Notably, in these studies of axillary and plexus injuries, direct suture vs. graft repairs showed minimal differences in outcome, which challenges the assumption that direct repair is always superior for incomplete injuries [32].

As an additional comment to the three studies discussed in this section, minimal differences were observed in outcomes for neuromas-in-continuity when treated by suture vs. graft repair technique. These findings may, therefore, contradict previous reports that anticipate better outcomes for suture repair of PNIs over graft repair [19].

### 6.2. Median Nerve

Median neuromas-in-continuity may be caused by trauma or chronic compression, and are often discovered/treated months after the initial trauma or onset of compression (e.g., persistent or recurrent CTS) [33]. Goals of surgery include removing surrounding scar tissue or restoring architectural anatomy and/or vascular supply to the affected portion of the nerve [33]. Depending on the level of involvement (number of fibers impacted) and length of the affected segment, various treatments have been described, including: neurolysis, excision and direct repair, or excision with subsequent grafting. While decisions between neurolysis, excision, and grafting are most often driven by extent of involvement, each of these may also include wrapping via muscle flaps, fat flaps, or synthetic wraps/conduits (Table 2). Despite improvements in repair techniques and adjunctive treatments, there is currently no consensus on the optimal treatment algorithm.

Dellon and Mackinnon utilized a pronator quadratus (PQ) flap as a treatment for seven patients who presented with painful median nerve in-continuity lesions. At 22 months after surgery, 6/7 patients had good or excellent results [34]. A subsequent report described nine median nerve neuromas-in-continuity at the level of the carpal tunnel treated with neurolysis and PQ muscle flap [35]. At final follow-up, 8/9 (89%) experienced pain relief, and 6/9 (67%) had resolved Tinel’s sign. Of note, thenar motor function was unchanged in this cohort [35]. Belmahi et al. performed teno-neurolysis with vascularized PQ muscle flap on 17 patients, all of whom had complete resolution of pain symptoms [36]. Utilizing the technique outlined by Dellon and Mackinnon, De Smet et al. performed a neurolysis and PQ muscle flap to treat a palmer branch of the median nerve neuroma-in-continuity. Prior to treatment, the patient (female, 63-years-old) had reported debilitating pain and tenderness. However, postoperative results were promising, as significant improvements in range of motion (ROM) and grip strength were noted, along with resolution of the patient’s pain symptoms [37].

Elliot et al. performed a prospective study in which twelve median and two ulnar neuromas-in-continuity were treated with teno-neurolysis followed by wrapping of the nerve elements in vascularized forearm fascia. Of the pain modalities assessed postoperatively (spontaneous basal pain, spontaneous spikes of pain, pressure pain, movement pain, hypersensitivity pain, and global pain) 8/14 patients had complete resolution of all modalities of pain, 2/14 had mild pain in one or two modalities, and 4/14 continued to have moderate or severe pain of at least one pain modality [38]. Another report analyzed outcomes for six patients who presented with constant nociceptive pain and dysesthesia due to wrist level median nerve neuromas-in-continuity. Treatment by external neurolysis and a flexor digitorum superficialis muscle flap was performed, with postoperative follow up at 6 months (2/6 patients) and 20 months (4/6 patients) finding reduction of spontaneous pain and dysesthesia in all patients [39].

Strickland et al. treated 58 patients with recurrent symptoms of wrist pain and dysesthesia due to recalcitrant CTS by neurolysis and covering of the damaged median nerve with a hypothenar fat pad flap (HTFPF). Postoperative follow up (mean of 33 months) demonstrated relief of pain/dysesthesia in 51/58 (89%) of patients. Improvement of 2PD from expanded to normal was observed in 21/58 patients but remained expanded in 5/58 patients. One patient, unfortunately, experienced expansion of 2PD from a normal preoperative value [40]. A subsequent report altered Strickland et al.’s surgical technique by performing microneurolysis (neurolysis with the addition of an operative microscope) with an HTFPF. In this study, 28 patients continued to suffer from recurrent CTS after previous surgical decompression attempts were unsuccessful. Reoperation with microneurolysis and HTFPF yielded favorable results, as Tinel sign that had been present in all patients preoperatively disappeared in 26/28 patients, average 2PD decreased from 7 mm (range 5–12 mm) to 6 mm (range 4–8 mm), and average grip strength improved from 11 kg to 20 kg [41].

In addition to these reports, Mathoulin has conducted multiple studies where painful recalcitrant CTS was treated by neurolysis and HTFPF [42,43]. A report by these researchers stated that 41/45 (91%) patients treated by this technique had complete resolution of pain, with clinical/EMG evaluation indicating good or excellent results in 41/45 (91%) of patients as well [42]. In a retrospective study, evaluation for 56 patients treated by the same technique for the same condition had pain disappear completely in 51/56 (91%) patients, as well as improvement of 2PD from abnormal to normal values in 50/56 (89%) patients [43].

**Table 2:** **Publications discussing median nerve in-continuity neuromas.** Major outcomes for each publication included in discussion of median nerve neuromas-in-continuity.

Study (Year)	Technique/Adjunct	Indication/Injury Pattern	Pain Outcome	Sensory/Motor Outcome	Functional/Satisfaction Outcome
Dellon and Mackinnon (1984) [34]	External neurolysis + pronator quadratus (PQ) muscle flap OR excision + nerve graft + PQ flap	Symptomatic median nerve neuroma/neuroma-in-continuity with pain	All patients treated for pain had good or excellent relief; one had recurrent pain only after new traumatic reinjury	Good sensibility recovery in cases where PQ flap provided a vascularized bed for the graft	All available patients returned to work or household duty (two with reduced capacity); high satisfaction including Workers’ Compensation cases
Adani et al. (2002) [35]	End-to-end repair + PQ flap (8); sural nerve graft + PQ flap (1)	Traumatic neuroma/painful scarred median nerve at distal forearm or wrist	Pain relieved in 8/9 (89%); Tinel’s resolved in 6/9 and decreased in 3/9	Postop sensory: S3+ in 6/9; thenar motor grades (M4–M5) largely unchanged	6/9 fully satisfied; 3/9 partially satisfied
Belmahi et al. (2002) [36]	External neurolysis + vascularized PQ flap	Persistent median nerve pain/neuropathy	100% complete pain resolution	(Not specifically quantified for discrimination)	Not reported
de Smet et al. (1997) [37]	Neurolysis + PQ flap (Dellon and Mackinnon technique)	Disabling neuroma pain of superficial radial nerve (3) or palmar cutaneous branch of median nerve (1)	Resolution of preoperative pain and Tinel’s sign (palmar cutaneous median nerve case)	Wrist flex/ext improved from 48°/44° to 65°/60°; normal grip and forearm rotation postop	Patient reported “excellent” satisfaction
Strickland et al. (1996) [40]	External neurolysis + hypothenar fat pad flap	Refractory median neuropathy/painful neuroma after carpal tunnel release	Relief of dysesthesia/paresthesia in 89%	2-point discrimination (2PD) stayed normal in 35/58; improved to <6 mm in 21/58; unchanged in 5/58; only one worsened	Grip strength ↑ 40–85% depending on workers’ compensation status; >80% returned to work (most to same job); median RTW ~10–13 weeks for manual laborers
Craft et al. (2007) [41]	Microneurolysis + hypothenar fat pad flap	Persistent pain/neuroma after prior carpal tunnel surgery	Pain completely resolved in 23/28; Tinel’s disappeared in 26/28	2PD improved to ~6 mm in revision carpal tunnel patients	Grip strength improved from 11 kg to 20 kg
Mathoulin et al. (2000) [42]	Neurolysis + pedicled hypothenar fat pad flap	Recalcitrant carpal tunnel syndrome/painful median neuropathy	Pain completely disappeared in 41; remaining patients mostly rated outcomes “excellent” or “good”	Nerve conduction normalized in most; EMG/clinical exam classified 49% excellent, 45% good, 4.5% average, 2% failure	Authors conclude hypothenar flap improves trophic environment and relieves pain in resistant CTS
Mathoulin (2015) [43]	Neurolysis + pedicled hypothenar fat pad flap	Refractory median nerve pain/neuropathy	Pain disappeared completely in 51/56 (91%)	2PD returned to normal in 50/56 (89%)	QuickDASH scores improved significantly
Gasse et al. (2009) [39]	External neurolysis + distally based flexor digitorum superficialis (FDS) muscle flap (vascularized coverage)	Debilitating neuropathic pain from traumatic or iatrogenic neuroma around the wrist	Spontaneous and percussive pain markedly reduced in all; 3/6 became pain-free	DASH ~17.1 in evaluable patients; full finger ROM preserved	5/6 satisfied or very satisfied; most returned to work at least partially
Uemura et al. (2020) [44]	Neurolysis + radial artery perforator adipose flap (vascularized adipofascial coverage)	Persistent median neuropathy with pain/tingling	VAS for tingling pain improved from 8.6 → 1.8; Tinel’s at palmar wrist disappeared in 4/7 and improved in 3/7	Wrist flex/ext improved from 120° → 139°; QuickDASH 55.2 → 21.4; Hand20 60 → 28.7; distal motor latency and CMAPs trended toward normal	Functional wrist motion restored; symptomatic relief in all patients
Elliot et al. (2010) [38]	Teno-neurolysis + vascularized forearm fascial flap (nerve wrap)	Scarred/tethered median nerve with chronic neuropathic pain	Complete pain resolution in 8/14; residual only mild pain in 2/14; 4/14 had persistent moderate/severe pain	Significant reductions in spontaneous baseline pain, movement pain, pressure pain; global pain improved	Overall patient-reported pain burden dropped across modalities
Yamamoto et al. (2014) [45]	Excision and autograft using vascularized lateral femoral cutaneous nerve within an ALT flap transferred to the wrist	Recurrent painful median neuropathy after carpal tunnel release and failed neurolysis/epineurial suture	Severe pain resolved completely; Tinel’s migrated distally then disappeared	Semmes–Weinstein improved 5.08 → 4.31; regained painless wrist/finger motion	No recurrence at 15 months
Lanzetta et al. (2000) [46]	Excision of painful palmar cutaneous branch of the median nerve (PCBMN) neuroma	PCBMN neuroma after carpal tunnel release, ganglion excision, or endoscopic CTR	Complete pain relief by postoperative day 4; no recurrent Tinel’s	No new paresthesia in flexor forearm distribution	All patients returned to work without impairment; all satisfied
Jeudy et al. (2014) [47]	Tissue expansion → en bloc neuroma excision → direct end-to-end repair	Chronic painful neuroma after complex multifocal median nerve laceration	Postop pain relief rated “good”; no spontaneous or movement-evoked pain	Sensation improved from S0 to protective S1; Semmes–Weinstein 6.65; 2PD ~20 mm; thenar atrophy persisted	Returned to work at 12 months
Suryavanshi et al. (case report) [48]	Neurolysis + processed nerve allograft wrapped in AxoGuard nerve protector, sealed with fibrin glue	Chronic neuropathic pain and weakness 20 months after bilateral carpal tunnel release + blunt trauma	Pain improved 7/10 → 0/10 at 1 year; Tinel’s improved	2PD improved >15 mm → ≤8 mm; grip strength 17 lb → 70 lb; recovered thumb opposition and full fist	Marked functional recovery and strength restoration at 1 year

Uemura et al. performed seven cases of revision surgery to alleviate symptoms of recurrent median nerve neuropathy by neurolysis and wrapping of nerve elements with a radial artery perforator adipose flap. At a mean follow up of 26 months (range 10–46 months), results were pleasing as Tinel’s sign completely disappeared or was relieved in all patients, and significant improvements were noted in visual analog scale (VAS), QUICKDASH, and Hand 20 scores [44].

Seven patients with painful neuromas of the PCBMN at the wrist level were treated by complete stripping of the palmar cutaneous branch containing neuroma away from the median nerve. All patients were satisfied with the procedure as it alleviated Tinel’s sign and paresthesia at the wrist, as well as the flexor aspect of the forearm [46].

Yamamoto et al. reported a case study in which a 70-year-old female patient presented with recurrent symptoms of severe pain, hyperesthesia, and right upper extremity anhidrosis after previous treatment attempts by carpal tunnel release (CTR) and epineural neurolysis combined with suturing had failed. Surgical exploration revealed a median nerve neuroma-in-continuity, and treatment proceeded via internal neurolysis, with the median nerve then being covered by an anterolateral thigh (ALT) flap that included a vascularized lateral femoral cutaneous nerve (LCFN). Postoperative results at 5 months were promising, as the patient’s severe pain had completely resolved. In addition, significant improvements were noted in Semmes–Weinstein (SW) score, and wrist and middle finger MCP, PIP, DIP joint ROM [45].

Three additional case reports provided description of operative techniques and outcomes for the treatment of median nerve neuromas-in-continuity. A 35-year-old female patient presented with wrist pain and sensorimotor deficits of the median nerve distribution after having punched through a glass window a few years prior. Operative treatment for neuroma-in-continuity consisted of neurolysis followed by the insertion of an AxoGuard nerve protector and allograft that was sealed by Tisseel fibrin glue. Postoperative results at 12 months revealed that pain score had dropped from 7/10 preoperatively to 0/10 postoperatively, along with improved Tinel’s sign, 2PD, and grip strength [48]. Another case report described the use of a distal anterior interosseous nerve (dAIN) graft to treat a 43-year-old female patient with diminished thenar function due to a neuroma-in-continuity of the median nerve. After resection of the recurrent motor branch (RMB) of the median nerve, dAIN was grafted into the RMB of the median nerve distal to the neuroma, resulting in restored thenar muscle function [49].

Lastly, a 42-year-old male presented with diminished sensory function, severe pain, and abductor pollicis brevis atrophy secondary to a median nerve neuroma-in-continuity. Surgical treatment consisted of 8 weeks of nerve elongation by silicone tissue expansion, followed by excision of the neuroma and direct end-to-end repair. Postoperative analysis at 2 years revealed significant reductions in pain and some improvement in sensory function. However, abductor pollicis brevis deficits remained uncorrected, and the patient was dissatisfied with the results [47].

### 6.3. Ulnar Nerve

A retrospective study by Kim et al. evaluated 127 cases of ulnar nerve in-continuity lesions, of which 87 recorded NAP and 40 were absent for NAP. Four different surgical techniques were utilized to treat these injuries, including internal/external neurolysis, split (partial graft) repair, excision and suture, or excision and graft. Successful outcomes were defined as achieving at least an LSUHSC Grade Level of 3 [50].

The results from Kim et al.’s report bring a few items into consideration. First, a high rate of success was noted for lesions in-continuity with NAP present when treated by neurolysis alone. Second, although only performed in three patients, the split repair technique for partially-transected nerves proved successful, regardless of the presence/absence of NAPs. Third, in-continuity lesions (absent NAP) treated with direct suturing had acceptable outcomes more often than those treated with a graft [50]. However, in contrast to this last point, two other reports have achieved acceptable results using a graft technique for ulnar nerve in-continuity lesion repair, albeit in rather limited sample sizes [34,51]. These findings serve as a reminder of the need for continuing research on suture vs. graft repair outcomes, especially in regards to in-continuity lesions.

### 6.4. Radial Nerve

The radial nerve stems from the posterior cord of the brachial plexus and supplies much of the innervation for the dorsal aspect of the arm, forearm, wrist, and hand. The posterior interosseous nerve (PIN) is a distal continuation of the radial nerve and is responsible for innervating much of this motor/sensory innervation in the posterior forearm [52]. Esquenazi et al. examined outcomes for iatrogenic injuries to the radial nerve in nine patients that resulted in-continuity lesions. Those with positive NAP were treated by external neurolysis, with eight patients achieving a Grade 4 and 1 achieving a Grade 5 functional recovery. On the other hand, eight patients with in-continuity lesions and negative NAP findings were treated by end-to-end suture (seven) or graft repair (one). Of these eight patients, five obtained a Grade 3 and three obtained a Grade 4 recovery, thus indicating that radial nerve in-continuity lesions are more likely to have better functional outcomes if positive NAP are present [53]. However, a report by Kim et al. evaluated outcomes for PIN in-continuity lesions that suggest no significant differences exist based upon the presence/absence of NAP. In this study, 28 patients with positive NAP were treated by neurolysis and all achieved a Grade of 3 or higher. Patients negative for NAP were treated by suture (two patients) or graft (five patients), and just like the positive NAP group, all obtained at least an LSUHSC Grade of 3 [54]. Esquenazi et al. and Kim et al.’s studies do provide evidence in support of positive outcomes for radial nerve in-continuity lesion repairs, but they also present contrasting data regarding the impact NAPs may have on postoperative outcomes [53,54]. Key studies discussing ulnar and radial nerve in-continuity neuromas are summarized in Table 3.

Murovic evaluated outcomes for 1837 upper-extremity nerve lesions based on lesion type. Lesion types were reported as sharp (complete transection, repaired within 72 h of injury), secondary (delayed) repair via suture or graft technique, in-continuity lesion with positive NAP treated by neurolysis, and in-continuity lesion with negative NAP and treated via suture or graft technique. Injuries were observed in the median, radial, and ulnar nerves; outcomes were reported in terms of the LSUHSC grading system, where a Grade of 3 or higher was considered a “good result” [55].

The results from Murovic’s study provide evidence for two important comparisons. First, positive outcomes were observed more often in primary repair than in secondary repair. This finding is consistent with several other studies that have been published [25,56]. Second, in-continuity lesions had better results than sharp lesions if positive NAP were present, but not when NAP were absent [55].

### 6.5. Digital Nerves

Surgical treatment for digital nerve neuromas-in-continuity may prove particularly challenging due to the complexities of operating on the smaller nerve elements and muscles of the hand [11]. As simple excision alone may not be sufficient to treat these injuries, treatment techniques that include resection and relocation into musculoskeletal tissue, nerve grafts, or various forms of nerve guides have been reported [11,12].

A retrospective study evaluated eight patients who had become disabled from work because of pain and hypersensitivity associated with digital neuromas-in-continuity of the hand or thumb. Treatment commenced by external neurolysis for all patients, followed by relocation to either the lumbricals (4/8 patients) or the intrinsic muscles of the thumb (4/8 patients). Postoperative evaluation at mean of 24 months demonstrated pain disappearance/relief for all patients, with 6/8 recommencing work activities [57]. A subsequent report described 14 painful digital nerve neuromas-in-continuity treated by resection and relocation into either bone or muscle tissue, with this procedure leading to successful resolution of pain in all patients [58].

Nunley et al. used the anterior branch of the medial antebrachial cutaneous nerve (MABC) as a graft to treat 21 digital neuromas-in-continuity, which yielded modest success for sensory healing. Follow up (average of 57 months) demonstrated that 20/21 patients regained ability to distinguish between sharp and dull stimuli. However, 2PD values varied from 5 mm (three patients), 6–10 mm (nine patients), 11–16 mm (six patients) and none (three patients). In addition, only 2/21 patients received a normal Semmes–Weinstein score postoperatively [59].

Malizos et al. treated 23 patients suffering from sensory loss, pain, or both due to 27 cases of sensory digital nerve neuromas-in-continuity. Treatment consisted of neuroma excision and bridging of the concomitant gap with a vein conduit graft. At follow-up, (mean of 31 months) subjective results showed promise, as 21/23 patients regained sensory function in the hand, and pain resolved in 17/18 patients. However, with respect to objective outcomes, SNAP and CV remained significantly decreased when compared to the contralateral, healthy hand in these patients [60]. A subsequent retrospective report by Thomsen et al. demonstrated favorable results for 10 painful digital neuromas-in-continuity treated operatively by excision and collagen conduits, as there were no complaints of recurrent pain at 12 month follow up. In addition, 5/10 digits had good (6–10 mm) or excellent (<6 mm) return of 2PD, and the average QUICKDASH score was 19.3 (0–39) [61]. However, the results of this study were not all promising, as five patients continued to have cold intolerance [61].

Building upon the work of these researchers, Foo et al. presented a case where an arterialized PIN graft was used to treat a digital neuroma in a 23-year-old male who had previously been treated unsuccessfully by primary repair for laceration of the ulnar digital nerve of the thumb. Postoperative outcomes measured at 1 year demonstrated resolution of pain and improvement in Semmes–Weinstein score from >6.65 (loss of sensation), to 3.61 (diminished protective sensation) [62]. Despite these positive outcomes, Foo et al. caution that outcomes of neurolysis, nerve grafting, or synthetic conduits currently remain unpredictable for digital nerve injuries [62]. Thus, there is need for continual exploration and study of these painful and potentially debilitating injuries in order to improve confidence in patient outcomes. Key studies discussing digital nerve in-continuity injuries are summarized in Table 4.

## 7. Timing and Mechanistic Considerations

### 7.1. Primary vs. Delayed Repair

As previously mentioned, a “wait-and-see” approach allows time for injured nerves to regenerate, and Omer has reported on multiple cases of spontaneous recovery of PNI [20].

When patients fail to recover with nonoperative treatment, surgical exploration is often indicated. Unfortunately, results of surgical intervention are best when performed immediately (within 72 h) after injury [19]. Given that acute repair leads to better functional restoration, delays resulting from a “wait-and-see” approach may cause lasting functional deficits [19].

For more severe injuries and in accordance with the principle that early repairs are better than late, Kim et al. recommend immediate surgical repair for sharp (total) transection injuries (e.g., glass, knife), or surgical repair 2–4 weeks after injury for blunt (total transection with significant soft tissue damage) injuries (e.g., chainsaw, lawn mower blade) [63]. Topuz et al. supported this recommendation with an emphasis that morbidity rates can be reduced by immediate surgical exploration, even without waiting for electrophysiological confirmation, if clinical evidence points to total transection of a nerve [64].

Although a much broader time frame, multiple studies have found better outcomes for repair of in-continuity lesions that take place within 6 months of injury, as opposed to later [51,65,66]. In fact, one study found that delays in recognition and treatment for PNI contribute to poorer outcomes and has even been the cause of litigation in past cases [64].

However, it should be noted that one retrospective study evaluated outcomes for surgical treatment of 56 symptomatic neuromas of the forearm or hand and found that time from neuroma diagnosis to surgery was not statistically significantly associated with QUICKDASH scores or pain grade [67]. Nonetheless, the majority of reports indicate that earlier surgical intervention is better than delayed operative intervention [51,65,66].

### 7.2. Traumatic Lacerations and High-Energy Injuries

Not all partial nerve injuries are benign stretching injuries; many occur in the setting of trauma with mixed mechanisms. For example, gunshot wounds or blast injuries can cause a combination of contusion, traction, and partial laceration to a nerve. These ballistic nerve injuries often produce a spectrum of damage along the nerve segment, from intact fascicles to completely disrupted ones, all within the same nerve trunk [1]. The shockwave from a bullet can contuse and tear nerve fibers without a clean cut, resulting in a neuroma-in-continuity that may have irregular areas of damage [1]. Such injuries pose significant challenges regarding when to operate and how aggressively to intervene.

A recent review on ballistic PNIs highlighted controversies in management, particularly regarding the timing of exploration and the role of neurolysis vs. resection [68]. The authors emphasized early surgical exploration for gunshot wounds with nerve deficits, as waiting too long can allow treatable lesions-in-continuity to degenerate further [68]. They propose an algorithm where if a nerve deficit is present after a ballistic injury, one should perform exploration and intraoperative assessment (including NAP testing) relatively early (in acute or subacute phase) [68]. If the nerve is in continuity and shows some conduction or continuity intraoperatively, a neurolysis might be attempted; if it is clearly nonfunctional, resection and grafting is recommended. For large nerve defects caused by high-energy trauma, grafting is generally required (sometimes multiple cable grafts), whereas smaller defects can be directly sutured (often achieved by mobilizing the limb or flexing a joint to reduce tension) [68]. In proximal injuries (e.g., brachial plexus or proximal sciatic), outcomes of grafting are poor when distances are long, so early nerve transfers should be considered to restore function [68].

Intraoperative electrical testing and inspection are crucial. In cases like injection injury to the sciatic nerve or a blast fragment injury to a nerve, early exploration has been shown to improve outcomes and even reduce neuropathic pain incidence. Direct end-to-end repair is preferred for sharp lacerations, even if one must transpose or flex joints to get a tension-free repair [68]. For blast injuries with segmental loss, nerve grafting and sometimes supplemental nerve transfers (to bypass the zone of injury) are indicated [68]. With timely and appropriate surgical management, even some severe combined-mechanism injuries can recover useful function, but without intervention, these injuries often result in lifelong deficits and neuropathic pain [68].

### 7.3. Blunt Crush, Traction/Stretch, and Contusion Injuries

Blunt crush and traction injuries typically produce axonotmesis within an in-continuity trunk, often with edema and hemorrhage that obscure healthy fascicular planes. A short early-subacute window (≈2–3 weeks) permits demarcation of nonviable tissue while avoiding prolonged denervation [9,69]. Exploration with NAP testing then distinguishes conductive lesions (amenable to external/intraneural neurolysis ± protective wraps or local vascularized tissue coverage) from nonconductive segments that require resection and tension-free repair or grafting [69]. Traction injuries with progressive weakness, severe dysesthesia, or evolving Tinel should be evaluated earlier, as clinical deterioration implies ongoing axonal loss despite continuity.

### 7.4. Injection, Thermal/Electrical, and Ischemic/Compartment Injuries

Chemical injection injuries (e.g., to the sciatic nerve) can cause profound intraneural necrosis disproportionate to external findings; early exploration to evacuate intraneural hematoma/necrosis and decompress tight fascicular compartments may improve outcomes and reduce chronic pain. Thermal injuries create coagulative necrosis that behaves like a segmental transection; debridement to healthy fascicles with repair/grafting is indicated [70]. High-voltage electrical injuries can produce deep intraneural damage and skip lesions; treatment mirrors high-energy trauma with an emphasis on early assessment beyond the apparent cutaneous burn [19]. In ischemic or compartment etiologies, urgent decompression (and fasciotomy when indicated) takes priority, followed by staged reassessment and reconstruction once tissue viability is clear.

### 7.5. Modifiers Affecting Timing and Strategy

Several factors refine timing decisions: (i) level of injury (proximal lesions require earlier action due to long regeneration distances); (ii) gap length and tissue quality (long gaps and scarred beds favor grafting and, if proximal/delayed, nerve transfer strategies); (iii) patient factors (age, diabetes, smoking, radiation, and crush-contaminated wounds slow regeneration and predispose to neuroma); and (iv) denervation biology (motor end-plates progressively lose receptivity over months; reinnervation potential falls as time to target lengthens) [19]. As a rule of thumb, lack of objective recovery by ~3 months (earlier for proximal/high-energy injuries) warrants exploration; by 6 months, in-continuity lesions without progress usually merit definitive neurolysis or reconstruction; beyond 9–12 months for proximal motor targets, nerve transfers or tendon transfers should be prioritized over lengthy grafts to maximize functional return [71].

### 7.6. Prognostic Indicators of Recovery

Across cohorts summarized above, three consistent patterns emerged: (i) A positive intraoperative NAP strongly predicts success with neurolysis, with approximately 70–90% of patients achieving at least antigravity strength (≥LSUHSC Grade 3) [30,32,50,53]. (ii) Early intervention—typically within 6 months when spontaneous recovery plateaus—correlates with superior motor recovery and pain relief [51,63,64,65,66]. (iii) Absent NAP usually necessitates resection and tension-free repair or grafting to obtain meaningful improvement [30,50,55].

## 8. Clinical Decision-Making Algorithm

**Figure 3:**
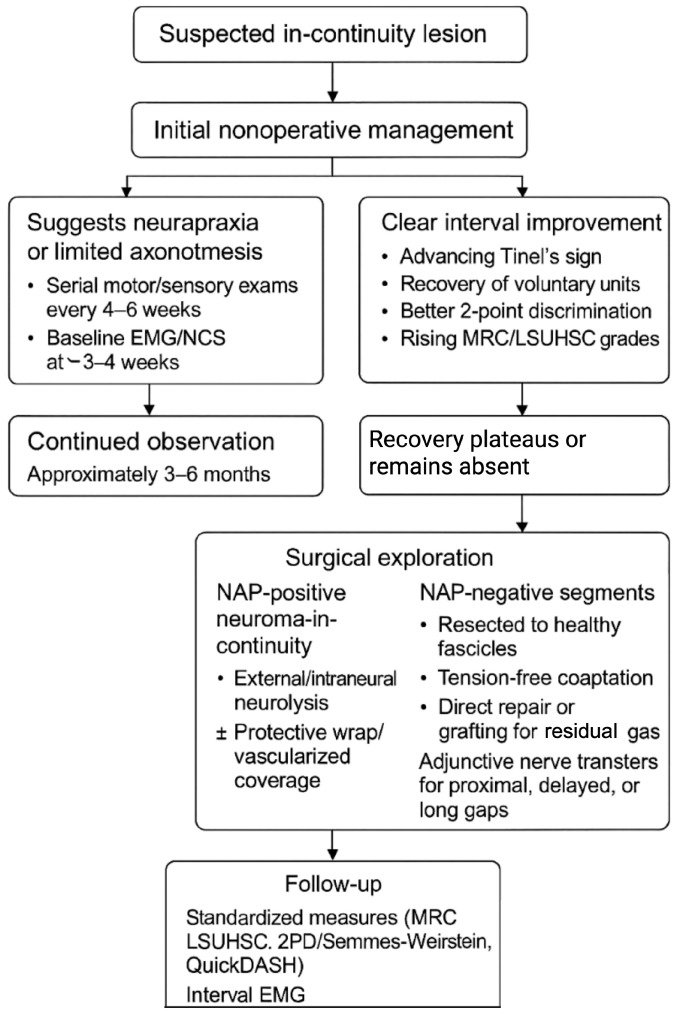
Clinical pathway for suspected in-continuity lesion.

For suspected in-continuity lesions, initial nonoperative management is appropriate when the presentation suggests neurapraxia or limited axonotmesis [17]. Patients should undergo serial motor/sensory examinations every 4–6 weeks with targeted therapy, and baseline EMG/NCS at ~3–4 weeks to document denervation and early reinnervation [2]. If there is clear interval improvement—advancing Tinel’s sign, recovery of voluntary units, better 2-point discrimination, or rising MRC/LSUHSC grades—continued observation is reasonable [21,22]. If recovery plateaus or remains absent by approximately 3–6 months (earlier for proximal or high-energy mechanisms), surgical exploration is indicated [23]. Intraoperatively, NAP testing across the lesion guides treatment: NAP-positive neuromas-in-continuity undergo external/intraneural neurolysis (±protective wrap/vascularized coverage), whereas NAP-negative segments are resected to healthy fascicles and reconstructed with tension-free coaptation (direct repair when feasible; grafting for residual gaps) [26,27,50]. For proximal, delayed, or long-gap scenarios, adjunctive nerve transfers may be considered to shorten regeneration distance [68]. Postoperatively, outcomes are followed with standardized measures (MRC/LSUHSC, 2PD/Semmes–Weinstein, QuickDASH) and interval EMG, with secondary procedures considered if reinnervation stalls. This clinical decision-making pathway is illustrated in Figure 3.

## 9. Future Directions and Advanced Techniques

Despite improvements in microsurgical repair techniques, outcomes after nerve injury—especially for high-grade or delayed repairs—remain inconsistent. As a result, researchers are exploring biological and technological adjuncts to enhance nerve regeneration and functional recovery [72]. Recently developed techniques include electrical stimulation (e-stim), fat grafting, and optogenetics.

### 9.1. Electrical Stimulation

Electrical stimulation, or “E-stim,” has been shown to accelerate axon outgrowth and recovery of twitch force, and add to tetanic tension and muscle action amplitude in multiple animal models [72]. Seminal preclinical studies, established that brief low-frequency electrical stimulation enhances axonal regeneration, accelerates target reinnervation, and improves muscle force recovery in rodent models of peripheral nerve injury [73,74,75]. These foundational data provide the mechanistic basis for subsequent human trials and highlight decades of experimental work supporting the translational use of electrical stimulation. These benefits have since been observed in human subjects with non-transected/partially-transected nerve injuries as well [76]. For example, a randomized controlled trial evaluated outcomes for two groups of patients based on whether or not they received e-stim following surgical decompression of the carpal tunnel for severe CTS. Follow-up at 12 months demonstrated that the experimental group (11 patients), which received low-frequency (20 Hz) e-stim for 1 h immediately after surgery, had significantly more motor units and increased motor/sensory nerve conduction in comparison to the control group (10 patients, no e-stim) [77]. Another randomized controlled trial assessed sensory recovery in 15 patients with completely transected digital sensory nerves treated by epineural repair and given 1 h of e-stim (20 Hz) prior to skin closure [78]. At 6 months follow-up, this group demonstrated significantly higher rates of normal 2PD (13/15 patients, 87%) when compared to a control group of 16 patients (7/16, 44%) that did not receive e-stim. The e-stim group also demonstrated better (3.33) QUICKDASH scores than the control group (19.42), although sample size prevented these values from reaching statistical significance [78]. Despite the evidence presented for the use of e-stim in these reports, studies regarding the use of e-stim as part of the surgical management of PNIs remain relatively few. Thus, more human subject research may be needed to determine how to best utilize this technology and confirm its ability to enhance peripheral nerve recovery [72].

### 9.2. Fat Grafting and Cell-Based Therapies

Fat grafting—often delivering adipose-derived stromal/stem cell (ADSC)-rich tissue—has been proposed to improve outcomes after operative treatment of PNIs [72]. Beyond serving as a vascularized, compressible cushion that limits adhesions around the repair, ADSC-containing grafts secrete pro-angiogenic and neurotrophic factors (e.g., VEGF, NGF, BDNF) that may modulate inflammation, reduce fibrosis, and support axonal elongation [72]. Multiple case reports/series describe pain reduction after excision of upper-extremity end-neuromas with adjunctive autologous fat grafting, and isolated reports in neuromas-in-continuity (e.g., De Jongh et al.) suggest potential sensory improvement when conservative measures fail [78,79].

From a translational standpoint, human evidence remains limited and heterogeneous. Techniques vary in harvest/processing (centrifuged lipoaspirate, micro-fragmented fat, “nanofat”), placement (wrap/overlay vs. interposition), and dose/volume; outcome measures (pain VAS, 2PD/Semmes–Weinstein, MRC/LSUHSC, QuickDASH) and follow-up intervals are inconsistent. Most reports lack controls and include small samples, so efficacy beyond scar-shielding and analgesia is uncertain [72,78]. Safety has been acceptable in small series (minor donor-site morbidity), but volume resorption, oil cysts/calcifications, and technique dependence are practical considerations. Use of expanded/culture-manipulated cells raises regulatory barriers that limit routine clinical deployment [72].

Autogenous (non-human) fat grafts may theoretically mitigate processing concerns; however, these have not been used clinically and show mixed results in animal models [72]. Because most published work evaluates complete transections, well-designed comparative studies in non-transected/partially transected PNIs are needed. Ideally, randomized trials would test neurolysis or resection-and-repair ± fat/ADSC wrap, use standardized pain and function endpoints, and follow patients ≥12 months to define indications, dosing, and additive benefit [72,78].

Additionally, a recent experimental study by Kahraman et al. demonstrated that vitamin B-complex therapy enhanced peripheral nerve regeneration and functional recovery in a rat model, with histologic evidence of improved myelination and reduced fibrosis and better electrophysiologic performance [80]. While mechanistically compelling, human data are lacking; pragmatic trials (e.g., perioperative B-complex supplementation as an adjunct to neurolysis or reconstruction) are warranted to define indications, dosing, and additive benefit.

### 9.3. Optogenetics

The last novel therapy for PNI discussed in this section is optogenetics. Optogenetics involves the use of gene modification to produce specialized channel proteins, “opsins”, which can be activated by specific wavelengths of light [72]. The stimulation of opsins can cause neuron depolarization and potentially assist in peripheral nerve regeneration [72]. For example, Park et al. stimulated the dorsal root ganglia of transgenic mice expressing a special opsin and found that the light-sensitive ganglia demonstrated significantly increased neurite growth in comparison to non-stimulated opsin and wild-type ganglia [81]. In addition to this report, Ward et al. observed positive effects on both motor axon function and muscle reinnervation of optically stimulated sciatic nerve neurons in mice [82]. Despite the positive results in these animal-based studies, optogenetics has yet to be employed in the treatment of PNIs in human subjects, leaving the clinical value of this modality undetermined [72].

### 9.4. Robotic Microsurgery

Robotics is emerging as a technical adjunct for peripheral nerve reconstruction, particularly in settings that demand extreme precision, deep or awkward exposure, or surgeon fatigue mitigation. Robotic microsurgical platforms offer several theoretical advantages: motion scaling (translating large hand movements into submillimeter instrument motion), tremor filtration, stable bimanual control, and three-dimensional high-magnification visualization [83]. In principle, these features can facilitate delicate neurorrhaphy, complex intraneural dissection, and cable graft inset in anatomically constrained corridors. They may also improve surgeon ergonomics during long reconstructions [84].

There are, however, important limitations. Robotic systems add setup time and cost, require specific instrumentation, and currently provide limited or no true haptic feedback. Loss of tactile feel is nontrivial in peripheral nerve surgery, where assessment of fascicular texture, perineurial integrity, and tension across a coaptation is often done by hand [85]. Learning curve and room configuration can also be barriers, particularly in urgent trauma scenarios. Early reports in microsurgical and microsurgical applications (e.g., lymphatic surgery, small-vessel anastomosis) suggest feasibility of suture work at very small diameters, but high-quality comparative data for peripheral nerve repair are not yet mature [86].

In the near term, robotic assistance is most likely to play a role in (i) elective, highly controlled reconstructions where setup time is acceptable; (ii) deep or difficult-to-access fields where traditional ergonomics are punishing; and (iii) microsurgical coaptation tasks requiring stable, precision [83]. As instruments and haptic feedback improve, and as operative times decrease with experience, robotics may transition from a niche adjunct to a reproducible platform for complex nerve reconstruction. Ongoing work should directly compare robotic versus conventional repair in terms of coaptation quality, operative efficiency, complication rates, and long-term functional recovery.

### 9.5. Autologous Nerve Transfers, Grafting Innovations, and Emerging Augmentation Strategies

Autologous nerve transfer and autograft-based reconstruction remain cornerstone options when direct end-to-end coaptation is not possible. Advances in microsurgical technique, graft processing, and biologic augmentation continue to refine these methods and improve outcomes. Autologous cable grafts, classically using the sural, medial antebrachial cutaneous, or great auricular nerves, provide viable scaffolds for axonal regeneration and are still considered the “gold standard” for bridging large gaps [87]. Recent experimental and early clinical work, however, has explored several strategies to optimize autograft efficacy.

Processed and decellularized autograft optimization: Improved intraoperative fascicular alignment using high-magnification mapping, fibrin-sealant coaptation, and biologic sheaths (e.g., collagen or umbilical cord allografts) have shown promise in reducing fibrosis and promoting directed axonal ingrowth. Refinements in decellularization and sterilization protocols for processed autologous grafts seek to preserve extracellular matrix architecture and neurotrophic signaling while minimizing immunogenicity, bridging the gap between autograft and allograft performance.

Vascularized and pedicled autografts: Vascularized autologous grafts (e.g., radial sensory, sural, or ulnar fascicular flaps) maintain intrinsic microcirculation, improving early revascularization and axon survival—particularly valuable in scarred or irradiated beds. Small-series human data suggest faster axonal conduction recovery and higher MRC strength scores compared with conventional non-vascularized grafts, though controlled trials are lacking.

Targeted nerve transfers: When long regeneration distances limit recovery potential, particularly in proximal or delayed injuries, nerve transfers can provide faster reinnervation by redirecting redundant donor fascicles to denervated targets. Contemporary examples include the Oberlin transfer (ulnar fascicle to biceps branch of musculocutaneous), double fascicular median/ulnar transfers for elbow flexion, spinal accessory to suprascapular, and intercostal to musculocutaneous transfers. In incomplete injuries, selective partial transfers can augment recovery while preserving donor function. Recent advances in intraoperative nerve mapping and ultrasound-guided planning have improved precision and minimized donor morbidity [88].

Adjunctive biologic and synthetic augmentations: Novel materials, including autologous platelet-rich fibrin, fibrin-based conduits, and nanofiber-aligned scaffolds, are being investigated to enhance Schwann cell migration and neurotrophic factor delivery at coaptation sites [89]. Early translational studies integrating autologous grafts with cell-laden hydrogels or ADSC-seeded matrices show increased myelination density and faster functional recovery in preclinical models.

## 10. Limitations

Relatively speaking, literature and information regarding outcomes for transection nerve injuries are abundant. Where information is lacking, however, is for the outcomes of surgically repaired non-transected and partially transected nerve injuries. Part of the reason for this limited data is accounted for by the studies that fail to differentiate outcomes for transected nerve repairs and partially-transected nerve repairs [63,90]. For instance, Secer et al. evaluated 2210 peripheral nerve lesions from gunshot wounds (GSW), all of which were repaired by direct suture, using nerve graft, or neurolysis. Lesions were classified as complete rupture (239), lesion interrupted by neuroma/fibrosis (508), partial rupture (191), neuroma-in-continuity (413), and intact nerves compressed only by fibrosis (859). Although this data was used to determine the influence of type of peripheral nerve (e.g., radial, median, ulnar, etc.) and injury level (e.g., upper arm, forearm, wrist) on postoperative outcomes, no findings were presented comparing the five different lesion type groups [91]. One other component that limited our study is the several articles cited that had small sample sizes, which may have inadvertently introduced sampling bias into study findings. Beyond limited sample sizes, the evidence is dominated by small, single-center retrospective series with heterogeneous outcome metrics, inconsistent follow-up, variable NAP protocols, and minimal adjustment for confounders (mechanism, gap length, injury level, age, timing), all of which introduce selection, measurement, and confounding biases and likely publication bias.

Additionally, preclinical work suggests that central nervous system plasticity following peripheral nerve injury, including maladaptive changes within the spinal cord and motor cortex, may contribute to incomplete functional recovery even after successful peripheral reconstruction. These central reorganization phenomena have been observed in rodent models (e.g., altered inhibitory interneuron activity and cortical remapping) and likely influence sensorimotor integration post-repair. Although human data remain limited, this underscores the importance of future studies integrating spinal cord and cortical-level analyses to fully elucidate determinants of functional recovery.

## 11. Conclusions

Peripheral nerve injuries (PNIs) are common and present with varying symptoms depending on the severity and nerves involved. Although available standard-of-care therapies are ineffective in consistently achieving full functional recovery, some trends have been identified from the literature. Information regarding outcomes for non-transected/partially transected PNI repair is limited—especially in the lower extremity—but findings are generally positive. Several studies have reported moderate success for surgically repaired upper-extremity lesions in continuity [30,31,32]. In practice, suspected in-continuity PNIs are observed with serial examinations and EMG; if recovery plateaus by ~3–6 months, exploration is undertaken, and intraoperative NAP then guides treatment. Several reports demonstrated better outcomes for patients with intact nerves that were treated by neurolysis; in some cases, even complete transection repairs had better results [30,31,55] Although these isolated results should be interpreted as trends rather than pooled effect estimates, they are notable. Given the current information available, it is difficult to account for why transected nerve repairs in some cases demonstrated better outcomes than in-continuity lesion repairs. One plausible theory is that the transected lesions may undergo repair sooner, and thus within an opportune window for treatment, as opposed to a period of nonoperative treatment and then delayed repair for in-continuity lesions [19]. Nonetheless, more studies are needed to not only determine outcomes for non-transected and partially transected nerve lesions, but also how to achieve the best possible outcomes for patients suffering from these painful and potentially debilitating injuries. Future work should prioritize multicenter prospective cohorts/registries with standardized reporting (MRC/LSUHSC, 2PD/Semmes–Weinstein, QuickDASH) and harmonized timing/NAP protocols, as well as pragmatic trials testing neurolysis or resection-and-repair ± adjuncts (e.g., brief electrical stimulation, fat/ADSC wraps, vitamin B-complex).

## Figures and Tables

**Table 1:** **Classification of nerve injuries.** Common causes, pathophysiology, surgical interventions, and expected recovery for various injury levels of the Seddon and Sunderland scales of nerve injury classification.

Seddon and Sunderland Classification of Nerve Injury		
Seddon	Sunderland	Injury
Neurapraxia	Grade I	Focal segmental demyelination
Axonotmesis	Grade II	Axon damaged with intact endoneurium
Axonotmesis	Grade III	Axon and endoneurium damaged with intact perineurium
Axonotmesis	Grade IV	Axon, endoneurium, and perineurium damaged with intact epineurium
Neurotmesis	Grade V	Complete nerve transection

Adapted from Seddon and Sunderland classification of nerve injury [1].

**Table 3:** **Publications discussing ulnar and radial nerve in-continuity neuromas.** Major outcomes for each publication included in discussion of ulnar and radial nerve neuromas-in-continuity.

Study (Year)	Technique/Adjunct	Indication/Injury Pattern	Pain Outcome	Sensory/Motor Outcome	Functional/Satisfaction Outcome
Kim et al. (2003) [50]	Internal/external neurolysis, split graft repair, excision and suture, or excision and grafting.	Persistent lesions of the ulnar nerve in-continuity.	Not reported	Lesions with NAP present showed high success with neurolysis alone; split repair successful in all three cases regardless of NAP; direct suture yielded better results than grafting for NAP-negative lesions.	Not reported
Rasulic et al. (2017) [51]	Internal/external neurolysis for in-continuity lesions, and nerve grafting or transfer for discontinuous lesions.	Iatrogenic peripheral nerve injuries, including those to the radial nerve, with either deficits in motor function or pain.	Not reported	Not reported	Satisfactory functional outcomes noted in 87.5% of in-continuity lesions and 81.8% of lesions not in-continuity.
Esquenazi et al. (2016) [53]	External neurolysis for NAP-positive lesions. Direct suturing or grafting for NAP-negative lesions.	Injection-induced radial nerve lesions resistant to spontaneous regeneration.	83.3% (5/6) patients experienced significant pain relief after neurolysis or neurectomy of forearm-level lesions.	LSUHSC Grade 3 or better recovery in patients receiving suture or grafting, and Grade 4 or better recovery in those receiving neurolysis.	Not reported
Kim et al. (2006) [54]	Neurolysis, primary or secondary suturing, or graft repair of in-continuity and not-in-continuity lesions.	Entrapments or tumors of the posterior interosseous branch of the radial nerve causing motor deficits.	Not reported	All but one patient (secondary graft repair) achieved LSUHSC Grade 3 or better motor outcomes.	Not reported
Murovic (2009) [55]	Primary repair within 72 h for sharp transections; secondary (delayed) repair via suture or graft; neurolysis for in-continuity lesions with positive NAP; suture/graft reconstruction for in-continuity lesions with negative NAP.	Large series of upper-extremity nerve lesions (median, radial, ulnar) categorized as sharp (acute primary repair), secondary/delayed repair, in-continuity +NAP (neurolysis), and in-continuity −NAP (suture/graft).	Not reported	Outcomes reported using LSUHSC grade (≥3 = “good”): primary repair > secondary repair; in-continuity +NAP > sharp; in-continuity −NAP < sharp (worst among the groups).	Not reported

**Table 4:** Publications discussing digital nerve in-continuity injuries.

Study (Year)	Technique	Pain Outcome	Sensory/Functional Outcome
Nunley et al. [59]	Excision + MABC nerve graft	Pain relief; regained sharp/dull sense	2PD: 5 mm (three), 6–10 mm (nine), 11–16 mm (six); two normal SW
Malizos et al. [60]	Excision + autologous vein conduit graft	Pain resolved in 17/18	21/23 regained protective sense; SNAP/CV ↓
Thomsen et al. [61]	Excision + collagen conduit reconstruction	No recurrent pain at 12mo	2PD ≤10 mm in 5/10; QuickDASH 19.3; cold intolerance 50%
Foo et al. [62]	Arterialized posterior interosseous nerve graft	Complete pain resolution	SW improved >6.65 → 3.61; regained protective sense

## Data Availability

No new data were created or analyzed in this study. Data sharing is not applicable to this article.
